# Global Size Pattern in a Group of Important Ecological Indicators (Diptera, Chironomidae) Is driven by Latitudinal Temperature Gradients

**DOI:** 10.3390/insects13010034

**Published:** 2021-12-28

**Authors:** Viktor Baranov, Jonas Jourdan, Blue Hunter-Moffatt, Sajad Noori, Simon Schölderle, Joachim T. Haug

**Affiliations:** 1 Faculty of Biology, Ludwig Maximilian University of Munich Biocenter, 82152 Planegg, Germany; schoelderle.simon@web.de (S.S.); jhaug@biologie.uni-muenchen.de (J.T.H.); 2 Department Aquatic Ecotoxicology, Institute for Ecology, Evolution and Diversity, Goethe University Frankfurt am Main, 60438 Frankfurt am Main, Germany; jourdan@bio.uni-frankfurt.de; 3 Department of Earth Sciences, Carleton University, Ottawa, ON K1S 5B6, Canada; bluehuntermoffatt@cmail.carleton.ca; 4 Centre of Natural History (CeNak), Leibniz Institute for the Analysis of Biodiversity Change, 20146 Hamburg, Germany; sajad.noori@studium-uni.de; 5 GeoBio-Center, Ludwig Maximilian University of Munich, 80333 Munich, Germany

**Keywords:** biogeography, body size, Diptera, functional traits, latitude, temperature size rule

## Abstract

**Simple Summary:**

The size of animals is a result of the complex interactions between the evolution of a group, the environment in which the animal lives, and its physiology. It has been known for a long time that warm-blooded animals (such as birds or mammals) become larger in colder climates. This phenomenon is called “Bergmann’s rule”, and it is caused by the necessity of the animals to produce and preserve their heat in colder climates. This is easier for larger animals, as they have a lower ratio of body surface area to body volume. In cold-blooded animals, such as insects, similar patterns have been found in some cases, but their origin is less clear. In this paper, we show a strong negative relationship between size and temperature in a large group of aquatic insects (non-biting midges). We found that wings of non-biting midges are shorter by 32.4 µm for every 1 °C of mean annual temperature increase. This finding is important for use of non-biting midges in monitoring aquatic ecosystem health and tracking global climate change.

**Abstract:**

Size is one of the most outwardly obvious characteristics of animals, determined by multiple phylogenetic and environmental variables. Numerous hypotheses have been suggested to explain the relationship between the body size of animals and their geographic latitude. Bergmann’s Rule, describing a positive relationship between the body size of endothermic animals and their geographic latitude, is especially well known. Whether or not insects exhibit a similar pattern has long been a subject for debate. We hypothesize that latitudinal size gradients are coupled to temperature variation affecting the metabolic rate of these merolimnic insects. We showcase a strong latitudinal size gradient in non-biting midges (Diptera: Chironomidae), based on the examination of 4309 specimens of these midges from around the world. Although phylogenetic position was a key predictor of wing length, we also found that wing length decreases by 32.4 µm per every 1 °C of mean annual temperature increase. This pattern was found across different taxa and could be detected in 20 of 24 genera studied. We discuss the reasons for this pattern origin and its palaeoecological implications.

## 1. Introduction

Numerous patterns have been established in the size distribution within different lineages of animals, driven by biogeography, phylogeny and ecology of these organisms [1,2]. Geographical patterns in body size distribution have received particular attention, with Bergmann’s Rule among the most famous of such patterns. Bergmann’s rule states that within a widely distributed monophyletic group of organisms, representative populations and species will increase in size from warmer to colder habitats [3]. Bears and penguins are two widely cited examples of Bergmann’s Rule. Both groups include species endemic to habitats that range from tropical to arctic and the average body size of these species increases with latitude [4].

Bergmann’s rule is traditionally accepted as occurring only among the endotherm, but ectotherm animals, such as insects, also exhibit similar size patterns, which can be more appropriately termed “temperature–size rule” [2,4]. Among insects, driving forces that have been implicated in producing inter- and intraspecific size differences include food availability, sexual selection, and predation, as well as temperature, latitude, and altitude and probably phylogeny [2,5,6,7,8]. Geographical clines of body size (hereafter “clines”) have been observed in many insect groups. The body size distributions of some of these appear to follow a temperature–size rule such as Bergmann’s rule with, on average, size increasing away from the equator [2]. However, for some groups, an opposite pattern was observed, or no correlation between body size and latitude at all [2,8]. For example, clines consistent with temperature–size rule have been recorded in numerous disparate species and small groups of insects, such as *Drosophila melanogaster* [9,10,11,12,13], *Musca domestica* [14], *Scathophaga stercoraria* [15], *Apis mellifera* [16,17], but also certain assemblages of Lepidoptera [18] and ants [19]. Examples for “inverse temperature–size” clines come from different butterflies [18,20], *Carabus nemoralis* and *Glossina palpilis* [15].

Ambient temperature of the environment is an important factor affecting growth rate and final size of ectotherm animals [21]. Several models have been proposed to explain potential clines of insect body size, including cell size of insects becoming smaller in warmer climates [2], temperature control of the duration of larval instars that triggers molting [2], and limited availability of oxygen in warmer climates [22]. Insects with aquatic larval stages (i.e., merolimnic insects) may be affected in their development in different ways than terrestrial insects [2,23].

The current acceleration of global climate warming means that understanding the impact of temperature on growth of merolimnic insects is increasingly important [24,25]. Recent research has shown that adult merolimnic insects, affected by elevated temperatures during development, grow to be smaller adults with shorter wings [23,24]. A widespread shift to smaller body sizes can negatively influence the dispersal ability of insects and may be one of several factors causing major changes in merolimnic insect populations [24].

Latitudinal gradients are excellent tools for investigating the direct and indirect effects of air temperature, examples of such gradients are observed in body size, metabolic rates and other physiological parameters [2]. Non-biting midges (Diptera, Chironomidae) are well suited as a model group to study latitudinal gradients in body size, due to their extremely wide distribution and high taxonomic and ecomorphological diversity [5,26]. Non-biting midges are mostly merolimnic and highly diverse [27,28], with over 7000 formally described species, and a global distribution from continental Antarctica to the Northern Polar circle [26]. Adult and immature stages of non-biting midges fulfill several different ecosystem roles—from carbon cycling to aquatic-terrestrial linkages [27]. Together, these factors make non-biting midges’ popular environmental indicators for both extant and late-Pleistocene-to-Holocene environments [26].

Several factors play an important role in determining adult body size of non-biting midges. For example, temperature [29] and number of generations per year [23] influence the size of non-biting midges. Previous studies have shown that, within several species’ groups (“genera”; i.e., *Pseudorthocladius* Goetghebeur, 1932), species occurring closer to equator were, on average, smaller than those who inhabited higher latitudes [5,30]. It is also possible that phylogeny is playing important role in the size distribution, with some groups simply having larger representatives on average [29]. While some mesocosm experiment have showed that Chironomidae might exhibit temperature–size rule (based on selected species only), no global analysis was ever conducted [23].

Here, we conduct the first large-scale analysis on geographical and phylogenetic patterns of adult body size of Chironomidae, aiming to identify global clines. We hypothesize to find an increasing wing length at higher latitude, which would be consistent with the assumption of the temperature–size rule. We further expect this trend to be evident over various taxonomic groups within the Chironomidae.

## 2. Materials and Methods

### 2.1. Data Acquisition

The analysis is based on a compiled dataset of 4309 specimens from 2090 species of Chironomidae. Measurements were obtained from published literature sources (1366 specimens/1123 species) and the Bavarian State Zoological Collection (ZSM Munich; 2943 specimens/967 species). We used wing length as a proxy for body length due to the abundance of published measurements for this structure, and the large number of available specimens with well-preserved wings. Due to the prevalence of males of non-biting midges, both in collections and published literature, we only measured the wing length of males. In cases of published specimens (*n* = 1366), we extracted measurements of the male holotype (normally right wing, if specified), as well as any other male specimens (paratypes, vouchers) of which place of origin has been specified Supplement File S2). We measured specimens of 971 species out of 2700 species in the ZSM collection (https://www.zsm.mwn.de/wp-content/uploads/2019/03/Chiro_Web-1.pdf, accessed on 1 September 2021). We measured all available wings of males, mounted on microscope slides. Species only represented by females, larvae or pupae were not considered.

**Figure 1:**
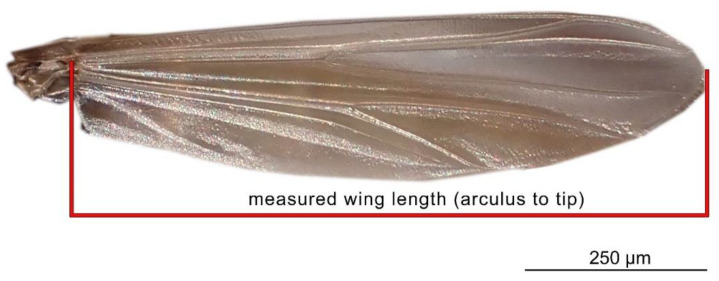
Wing of a male representative of a species of Tanytarsini (*Tanytarus* sp.) and the scheme of wing length measurement, applied in this paper. Photo by V. Baranov.

**Figure 2:**
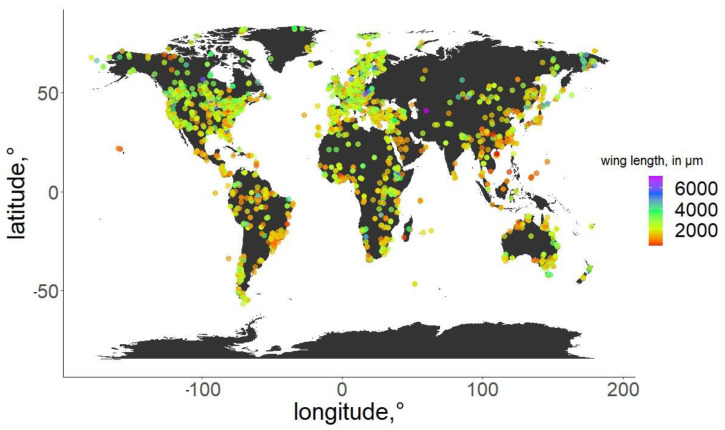
Geographic distribution of the specimens used in the analysis. Wing length of the specimens is color-coded.

For the specimens from the ZSM, we photographed the right wing of each specimen and measured it directly. In cases when the right wing was damaged, the left wing was photographed and measured instead. Images were taken using a DCM 510 ocular camera in conjunction with a Leitz Diaplan Microscope, using ×2.5 magnification, to nearest 10 µm, to capture the entire wing in a single image. All ZSM specimens measured were previously mounted on permanent microscope slides, either in Canada Balsam or Euparal. Wing length was measured as per standard procedure [31]: from the arculus sclerite proximally at the wing and the outermost point of the distal end (Figure 1). Measurements were taken from photos using Fiji (ImageJ public domain [32]). The geographic distribution of the measured specimens, and their wing lengths, are depicted in Figure 2. Geographic coordinates were either taken directly or estimated (from the geographic data provided) from labels or descriptions (when available). The database in its entirety is available as supplementary File S3. For every measured specimen, we extracted the geographical coordinates at which it was collected. Coordinates were taken either as provided by the authors of the published works, or inferred from the labels of the permanent slides, using Google maps, when necessary.

### 2.2. Data Analysis

Statistical analysis of the data was performed in R version 4.1.2 [33]. All R packages used are the latest available versions accessed on 1 December 2021. The R code utilized is accompanying this paper as Supplementary Material (Supplementary File S1–S9). To examine the relationships between the wing length and several independent variables thought to be important predictors of wing length, we constructed generalized linear squares (gls) models using the “nlme” package [34]. We used wing length, unpartitioned by species, as a response variable. We used the following independent variables in our model: (1) mean annual temperature (at 5 min of latitude resolution), extracted from the WorldClim database (https://worldclim.org/, accessed on 28 November 2021); (2) hemisphere (North or South) in which specimens were collected; (3) taxonomic identity (i.e., genus) of the specimen collected, and (4) interaction terms of these factors. For the analysis, we did not distinguish between the northern and southern latitudes in the latitude variable itself, using the additional hemisphere variable instead. We examined a possibility of running the same models with temperature as a predictor instead of the latitude. We repeated the same analysis with temperature as a predictor variable. Interaction between temperature and genera was removed due to the singular fit of the high-level random effect. We ran the gls model on the same subset of the genera, which were later for calculation of the Theil-Sen’s slopes. To account for the temporal autocorrelation in the data, we specified the correlation structure using the corAR argument in the gls function. We used the Akaike (AIC) information criterion to determine the optimal fit model.

No major violations of model assumptions (e.g., normal errors distribution and homoscedasticity) were detected in any of the models. Model results are reported as ANOVA tables, using the ANOVA function in the “car” package [35] and as the standardized effect sizes [36]. Owing to the non-normal distribution of the errors, when the overall dataset was split into the numerous multiple datasets by genus, we used nonparametric Mann–Kendall test to detect the trend and direction of changes in size over the latitudinal and temperature gradients. To access a rate of change in wing size over the latitude/temperature, we applied a nonparametric Theil-Sen’s slope estimator, using the mblm function in the “mblm” package [37]. To investigate whether larger wing length are leading to the larger shift is purely an artifact, we also log transformed (log10) the wing length and standardized it by adding 1 to every value. For an analysis of within-genus trends in wing length trends from temperature, we only used the genera with the numerous (>60 per genus) specimens measured. Therefore, the analysis of the within-genus trends was conducted on a reduced dataset with 2643 specimens, 1115 species.

Additionally, we ran a gls model with a Theil-Sen’s slope as a response variable and mean wing length per genus as an explanatory factor. Theil-Sen’s slope in this case is a measure of the rate of change of the mean wing length per genus in the latitudinal gradient.

### 2.3. Phylogenetic Analysis

To estimate the role of the phylogeny in the size distribution, we conducted an analysis of the phylogenetic correlation using the phylosig function of the “Phytools” package [38]. Due to the absence of a comprehensive phylogeny covering all the species examined in our analysis, we used the phylogeny from Cranston et al. [39] as a basis for computing the phylogenetic correlation. Therefore, our phylogenetic correlation analysis only represents a subset of 203 species of the main dataset. We analyzed a phylogenetic signal on the consensus tree [39] based on the consensus neighbor-joining tree, based on 100 bootstrap replications. We attempted to build a phylogeny for the larger proportion of the species studied, by automatic search of the matches of the sequences in the GenBank, but since we only had found a single gene sequences, cytochrome c oxidase subunit I, available for most species in the dataset, we decided to discard the idea of building a new multi-gene tree for all the examined species.

## 3. Results

**Figure 3:**
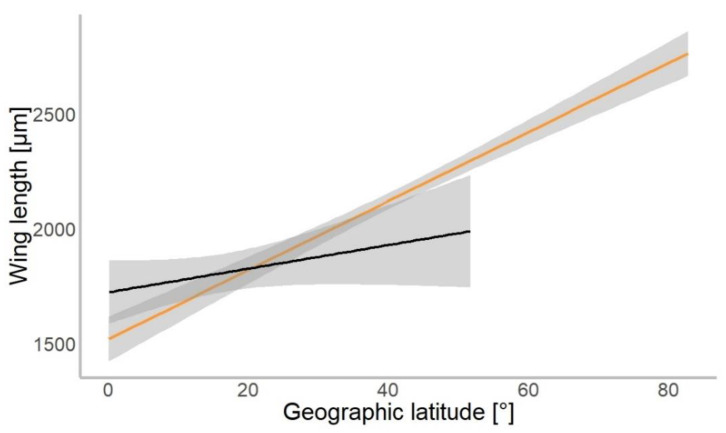
Chironomidae wing length across the latitudinal gradients. Orange line shows distribution of wing length in the Northern, and black in Southern hemisphere (for all data points plotted, see Figure S1).

Based on 4309 specimens of Chironomidae, we found a median wing length of 1.9 mm (±0.9 mm SD). In our gls model, the geographical latitude at which each specimen was collected was a significant predictor of the mean wing length (χ^2^ = 534.01, *p* = 0.001). Wings were getting longer further away from the equator in both hemispheres (Figure 3). The mean wing length of non-biting midges increased by 16.8 µm for every 1° of latitude away from the equator (median-based linear model, residual standard error: 867.7, *p* < 0.001, see Table 1). The genus (χ^2^ = 4309.88, *p* < 0.001) and interaction of latitude and genus (χ^2^ = 154.49, *p* < 0.001) as well as interaction term of hemisphere with latitude (χ^2^ = 4.62, *p* = 0.031) were significant predictors of wing length. The hemisphere from which the specimen originated (North or South; χ^2^ = 0.57, *p* > 0.05) was not a significant predictor of size.

In another model, we substituted latitude by the mean annual temperature for the coordinates from which specimen came. In this gls model, the mean annual temperature was a significant predictor of the mean wing length (χ^2^ = 548.52, *p* = 0.001). The genus to which a specimen belongs had a significant effect (χ^2^ = 4107.34, *p* = 0.001), as well as interaction of mean annual temperature with hemisphere (χ^2^ = 6.11, *p* = 0.01; see Table 2). Hemisphere had no effect on the wing length (χ^2^ = 3.26, *p* = 0.07). The mean wing length of non-biting midges, decreased by 32.4 µm for every 1 °C of temperature increase (residual standard error 870.5, *p* < 0.001). The Theil-Sen’s slope illustrates the significant interaction between mean annual temperature and wing length within genus. As such, 20 out of 24 genera showed decrease in the wing length with the increase of mean annual temperature, two genera showed increase (*Chaetocladius* and *Corynoneura*) and two more genera showed no significant pattern (genera *Orthocladius* and *Micropsectra*; Figure 5).

To examine the effect of taxonomic identity (i.e., genus) on the mean wing length, we investigated the relationship between the difference in wing length per 1° of latitude (Theil-Sen’s slope) and the mean wing size per genus. Using gls model, we found that mean wing size per genus was a significant predictor of the Theil-Sen’s slope (χ^2^ = 8.115, *p* = 0.004). However, when we log-transformed and standardized the wing lengths, no significant relationships were detected any more (χ^2^ = 0.7, *p* = 0.5; Supplementary Figure S2).

We analyzed our data for a possible phylogenetic signal for size distribution within Chironomidae, using the tree from the Cranston et al. (2012). Lambda of the phylogenetic signal was 0.9 with *p* < 0.001. Analysis has shown that representatives of certain ingroups (genera) of Chironomidae are much larger than the group average of Chironomidae as a whole (Supplementary Figure S1). Most of the specimens examined had on average a wing length from 2 to 4 mm (Supplementary Figure S1). Representatives of Aphroteninae had wings considerably shorter than 2 mm, while representatives of *Kiefferulus*, *Chironomus* and *Axarus* were considerably larger than average (Supplementary Figure S3).

## 4. Discussion

### 4.1. Phylogenetic Signal in Size Distribution within Chironomidae

**Figure 4:**
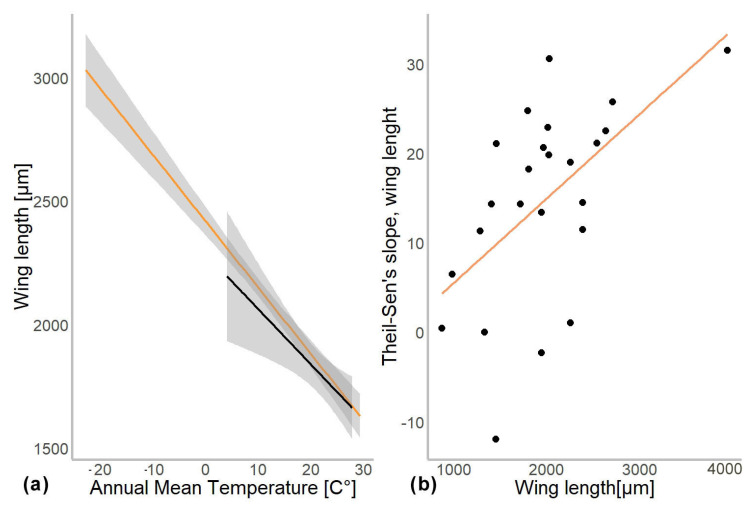
(**a**) Chironomidae wing length across the temperature gradients. Orange line shows distribution of wing length in the Northern hemisphere, and black in Southern. (**b**) Changes in wing length along latitudes (expressed as Theil-Slope) in relation to the average wing length of the genus (un-standardized length).

**Figure 5:**
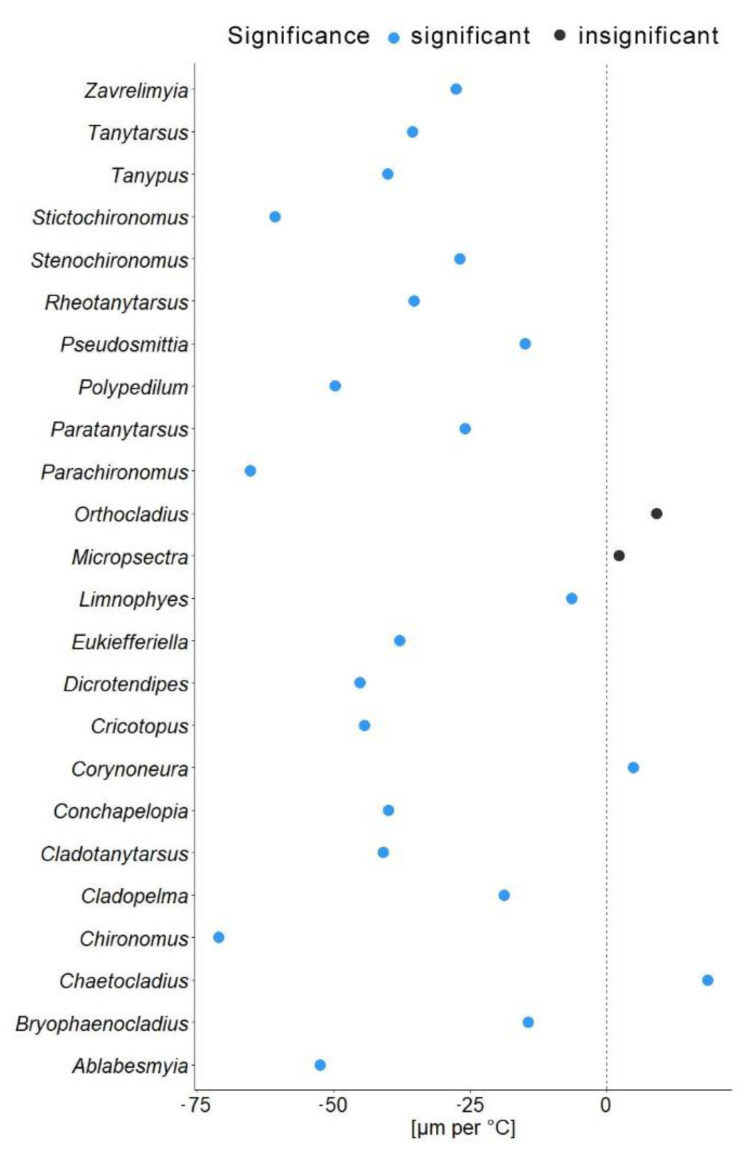
Theil-Sen’s slope of mean wing length shift per 1 °C. Blue dots indicate significant changes; black dots represent non-significant changes.

Our analyses demonstrated that genus affiliation was the strongest predictor of Chironomidae wing length (Table 2). This was also reflected by a strong correlation between phylogenetic structure and size distribution, indicating a strong historical component in the size distribution (Supplementary Figure S2). We have found a significant decrease in the in the wing length with increase in the mean annual temperature (Figure 4a). Despite a strong phylogenetic signal, we also found a clear trend of wing length increase with increasing distance from the equator in most groups (Figure 5). That means, both, representatives of genera with on average small and large representatives, grow larger in colder climates. Most genera thus follow temperature size clines according to Bergmann’s rule (i.e., a negative relationship between size and temperature). Only genera *Corynoneura* and *Chaetocladius* showed inverse pattern, while the genera *Orthocladius* and *Micropsectra* showed no significant trends in size at all. We currently lack an explanation of why *Corynoneura* and *Chaetocladius* did not follow the general pattern. *Corynoneura* is among the Chironomidae genera with the smallest specimens on average. Median wing length of *Corynoneura* specimens in our dataset was only 841 µm vs. median wing length for all the Chironomidae representatives in dataset of 1950 µm. It is well documented that small insects flight functions differ from the flight mechanics of the larger insects [40]. Since smaller insects experience the air as a much denser medium than their larger relatives, tiny *Corynoneura* are probably under different constraints of the wing size compared to their larger relatives [40,41]. That might have led to the reverse temperature–size rule in *Corynoneura*. No such clear-cut explanation can be suggested for the *Chaetocladius* representatives. They probably buckle the trend, as representatives of the genus were relatively underrepresented in our samples in comparison to the large global species diversity of the genus [28]. It is also possible that, due to the small size of *Corynoneura* representatives, no clear trend was revealed, as wing length was only measured to the nearest 10 µm. It is unclear why two other genera have shown no significant pattern at all. Additional material from these genera is required to elucidate this question.

### 4.2. Global Trends in the Wing Length of Chironomidae

As hypothesized, we found a strong negative temperature–size rule in Chironomidae. We found this pattern not only when we considered the entire group Chironomidae, but also within most major ingroups (genera). The general pattern of the latitudinal size distribution goes from smaller at equator toward larger away from it. This pattern was observed in both hemispheres, although the southern hemisphere is undersampled relative to the Northern hemisphere [27,28,42]. Additionally, sampling of Chironomidae in the Southern Hemisphere is biased toward several specific ingroups, such as Podonominae, Heptagyini [43].

### 4.3. Possible Drivers of Temperature–Size Rule in Chironomidae

Temperature appears to be one of the main drivers behind the size of adult non-biting midges [23,26,29] and the latitudinal size clines. The relationship between wing length and temperature seems to be mediated by: (1) the duration of ontogeny [29]; (2) the duration and number of the generations per vegetation season (voltinism) [23]; (3) the metabolic rate of a specimen [22]; (4) the oxygen availability in water [2,22,23]. Wonglersak et al. (2021) [23] showed that voltinism is a primary mechanism generating the negative relationship between adult size of the several examined species of non-biting midges and temperature. They found that multivoltine species with several generations per year tend to become smaller in higher temperature environments, while uni- or merovoltine species (with one or fewer than one generation per year) tend to become large with higher temperature or show no relationships at all [8,22,23]. This relationship between the number of generations per year and adult wing length can be the result of increased scarcity of resources and partitioning of nutrients for the more numerous larvae of multivoltine species, particularly in species which larval cohorts are overlapping temporally. Under higher temperature conditions, some of the multivoltine species produce more generations of larvae per year, increasing the scarcity of resources and leading to smaller average size when the larvae reach their adult forms [2]. Unfortunately, it is currently impossible to test this hypothesis on the global scale, due to the limited availability of the phenological and bionomic data for Chironomidae representatives outside Europe and North America [26]. Even when bionomic data are available, we lack information on the regional variation of phenology within the same species over the large distribution areas [26].

Additionally, Chown and Gaston [2] suggested that reduced oxygen solubility at higher water temperature can be a driver of the negative temperature–size relationship in non-biting midges. They hypothesized that larger species of non-biting midges will exhibit a more drastic decrease in size in higher temperatures as they require more oxygen during their larval development. Since oxygen solubility in water drops significantly with increasing temperatures, it will have a greater impact on species with larger larvae due to their higher oxygen demands [23,44,45,46]. This is consistent with the hypothesis of “aerobic scope protection”, which postulates that aquatic ectothermic animals reduce their food intake under warmer conditions to reduce oxygen consumption, which in turn leads to smaller wing length [23,45]. We did not find any evidence of aerobic scope protection in our data. Only uncorrected size data pointing to steeper Theil-Sen’s slopes in larger midges. When data were corrected (log transformed), this tendency disappeared. We think that absence of the trend in corrected data reflects a mathematical artifact, where larger animals merely have a larger absolute increase of the wing length in latitudinal gradient than smaller ones.

Therefore, it is unlikely that aerobic scope protection is the sole mediator of the relationship between the growth of aquatic ectotherm animals and the temperature of their environment [44]. As Hoefnagel and Verberk [45] showed, only under hypoxic conditions will the temperature–size rule manifest in ectothermic aquatic isopods (*Asellus aquaticus*).

### 4.4. Implications of the Findings for the Palaeoecological Analysis

Non-biting midges (Diptera, Chironomidae) remnants preserved in the sediments from the Pleistocene Holocene (11,650 Years BC–Present) are frequently used to reconstruct temperature patterns and climates of the past [29]. Majority of the Holocene and Pleistocene Chironomidae representatives, still exist in extant fauna which allows to reconstruct their climatic tolerance, using nearest living relative approach [29,47]. Reconstruction of the climate tolerance and ecology of the extinct animals is much less efficient proposition, and it’s getting less and less efficient the deeper we go back in time [47]. That is to say, fossil record of Chironomidae representatives from Jurassic is much less usefully for paleoclimate reconstruction than one from Pleistocene. We will struggle to extrapolate ecology and thermal preferences of Jurassic midges from their living relatives, as those will be quite distant, both phylogenetically and ecologically.

We think that a strong size–temperature rule detected here in Chironomidae can be used for physiognomic paleoclimate analysis. Physiognomic analysis is basing climate reconstruction on correspondence between morphological characteristics of the organisms and certain environmental variable [47]. We think that correspondence between Chironomidae wing length and temperature can provide a gateway for development of the trait library and software for analysis of the paleoclimate, based on Chironomidae fossils. Such an analytic framework can be organized in a manner similar to the CLAMP (Climate-Leaf Analysis Multivariate Program) [47]. Analysis of the paleoclimates based on Chironomidae, can be quite fruitful as there are hundreds of deposits containing Chironomidae fossils, spanning last 245 million years, thus providing wealth of material for analysis [48]

## 5. Conclusions

In conclusion, we found strong evidence for macroecological patterns in inter-specific wing length decline toward the equator. It appears that availability of food and rate of growth, mediated by temperature, are among the main reasons for the presence of latitudinal size clines following the temperature–size rule within the Chironomidae [22,26,29]. Present findings are contributing to our understanding of relationships between Chironomidae representatives and climate. This is important because Chironomidae are frequently used as climatological proxies, especially in palaeoecological reconstructions for the Holocene and late Pleistocene [29]. Understanding the relationships between Chironomidae representatives wing length and temperature might open new avenues in application of Chironomidae in palaeoclimatological studies. Among others, use of the Chironomidae size as a taxonomy-independent proxy for the paleotemperatures might be possible.

Future research should continue to identify underlying mechanisms, for example, by conducting common-garden studies where different species from various latitudinal origins are reared under laboratory-controlled environments. Understanding drivers that shape macroecological patterns and the evolution of clines is essential to predict dynamics and adaptation considering climate change.

## Figures and Tables

**Table 1:** ANOVA of the best fit gls model on wing length of Chironomidae. * denotes level of significance (* </ = 0.05; *** </ = 0.001).

Independent Variable	df	χ^2^	*p* Value (χ^2^)
Latitude	1	534.01	<0.001 ***
Hemisphere	1	0.57	0.44
Genus	24	4309.88	<0.001 ***
Latitude × Genus	24	154.49	<0.001 ***
Latitude × Hemisphere	1	4.62	0.03 *

**Table 2:** ANOVA of the best fit gls model on wing length (with temperature). * denotes level of significance (** </ = 0.01; *** </ = 0.001).

Independent Variable	df	χ^2^	*p* Value (χ^2^)
Temperature	1	548.5266	<0.001 ***
Hemisphere	1	3.2669	0.07
Genus	24	4107.34	<0.001 ***
Temperature × Hemisphere	1	6.11	0.01 **

## Data Availability

Data are contained within the article or attached as electronic supplements to this paper.

## Supplementary Materials

The following are available online at https://www.mdpi.com/article/10.3390/insects13010034/s1, Figure S1: (a) Chironomidae wing length across the latitude gradients. Orange line and points shows distribution of wing length in the Northern hemisphere, and black in Southern; (b) Same but with temperature as a predictor, Figure S2. Changes in wing length along latitudes (expressed as Theil-Slope) in relation to the average wing length of the genus (un-standardized length), Figure S3: Distribution of the wing length over the dated phylogenetic tree of Chironomidae (tree from Cranston et al., 2012). Tree is split in two parts to enhance the readability and comprehension. P1 and P2 are denoting parts of the tree 1 & 1, tree root is in a part 2, red circle and an arrow are denoting a place of tree separation, File S1: r_code—R code for the paper, Files S2–S6: c_cadi_dna, c_cadiv_dna, c_coi_dna, c_18s_dna and c_s28_dna—DNA alignments for the phylogenetic analysis. All DNA data based on the Cranston, P. S., Hardy, N. B., & Morse, G. E. (2012). A dated molecular phylogeny for the Chironomidae (Diptera). Systematic Entomology, 37(1), 172–188, File S7: cranston_names.csv—discrete size data for the phylogenetic analysis, File S8: main.txt—main database file/main file on which R analysis is based, File S9: df_temp.txt—file for the R analysis containing, in addition to the Chironomidae size data, mean annual temperature data from the (https://worldclim.org/, accessed on 28 November 2021).
