# Supplementary figures and images for: Global Size Pattern in a Group of Important Ecological Indicators (Diptera, Chironomidae) Is driven by Latitudinal Temperature Gradients
Source: Insects. 2021 Dec 28;13(1):34. doi: 10.3390/insects13010034 (PMC8781536; doi:10.3390/insects13010034)

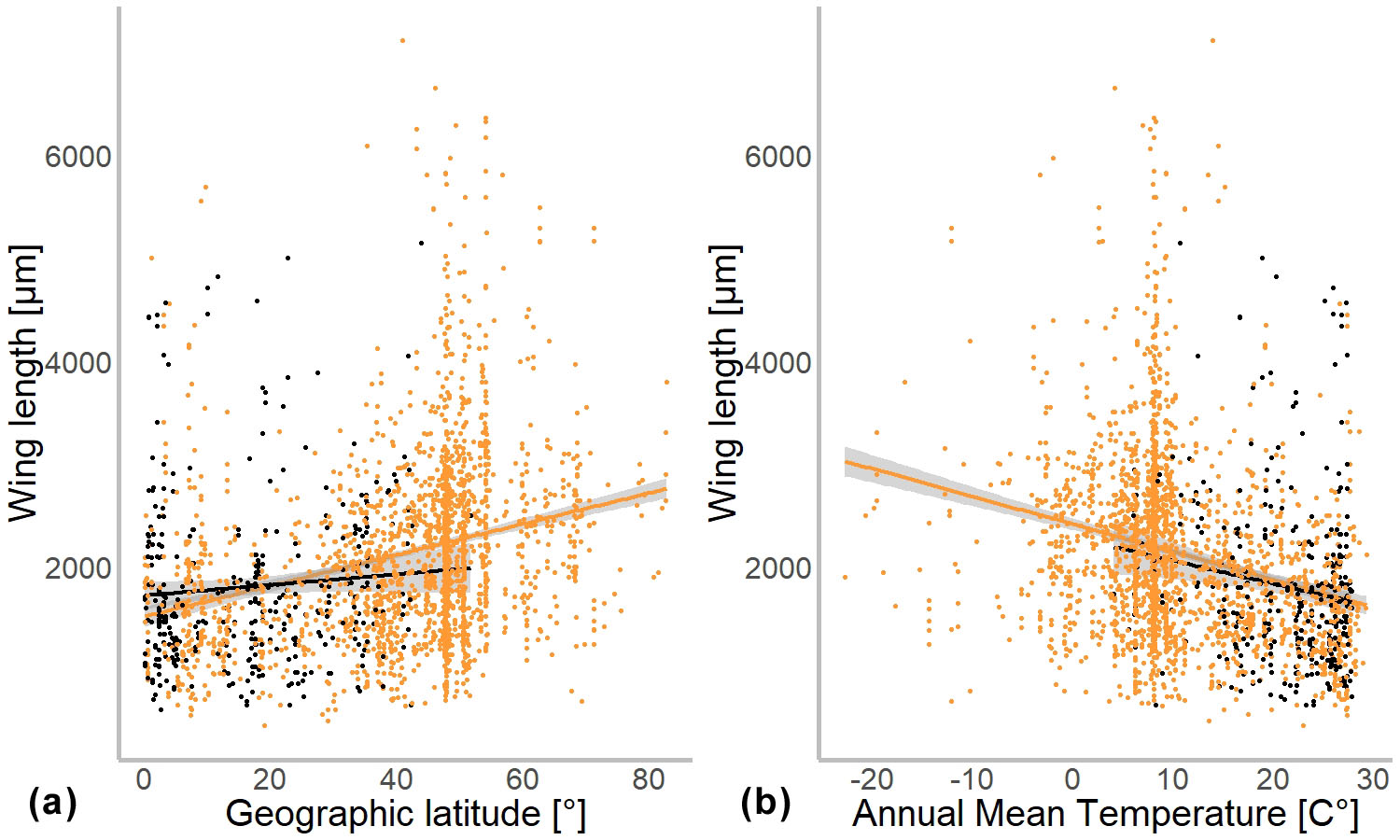

Supplement: Supplementary file 1 [file insects-13-00034-s001.zip › Figure S1.jpg]

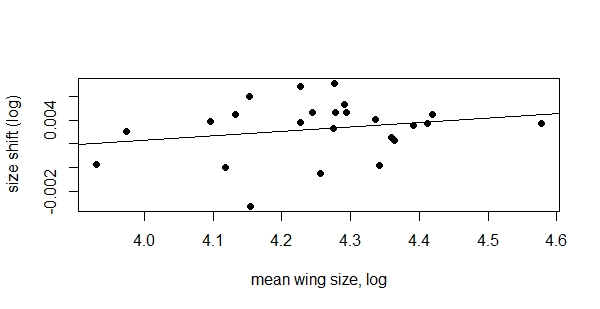

Supplement: Supplementary file 1 [file insects-13-00034-s001.zip › Figure S2.jpeg]

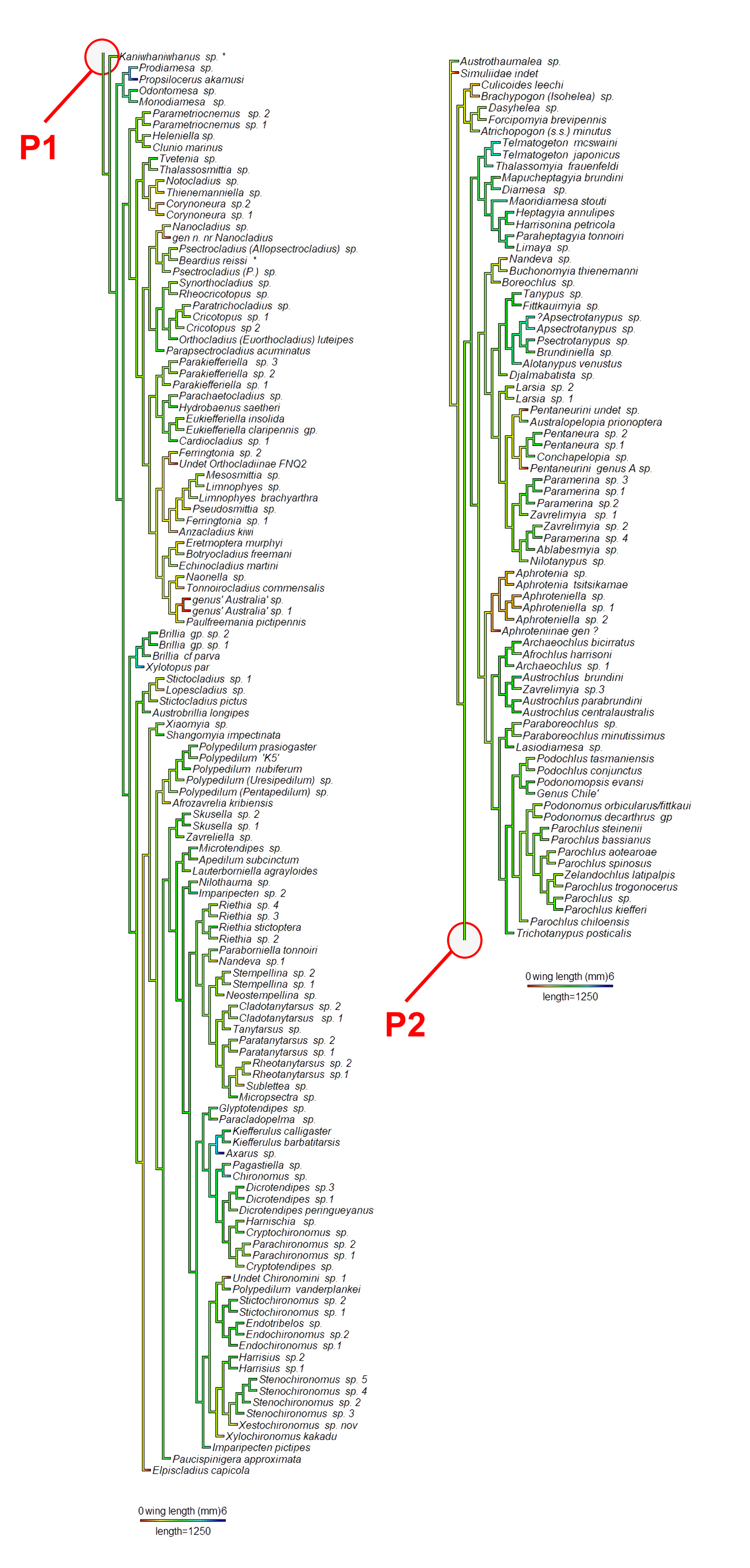

Supplement: Supplementary file 1 [file insects-13-00034-s001.zip › Figure S3.jpg]
